# Prediction of Device Characteristics of Feedback Field-Effect Transistors Using TCAD-Augmented Machine Learning

**DOI:** 10.3390/mi14030504

**Published:** 2023-02-21

**Authors:** Sola Woo, Juhee Jeon, Sangsig Kim

**Affiliations:** Department of Electrical Engineering, Korea University, 145 Anam-ro, Seoul 02841, Republic of Korea

**Keywords:** feedback field-effect transistors, machine learning, random forest regression, technology computer-aided design (TCAD), TCAD-augmented machine learning

## Abstract

In this study, the device characteristics of silicon nanowire feedback field-effect transistors were predicted using technology computer-aided design (TCAD)-augmented machine learning (TCAD-ML). The full current–voltage (*I-V*) curves in forward and reverse voltage sweeps were predicted well, with high R-squared values of 0.9938 and 0.9953, respectively, by using random forest regression. Moreover, the TCAD-ML model provided high prediction accuracy not only for the full *I-V* curves but also for the important device features, such as the latch-up and latch-down voltages, saturation drain current, and memory window. Therefore, this study demonstrated that the TCAD-ML model can substantially reduce the computational time for device development compared with conventional simulation methods.

## 1. Introduction

Recently, the prediction of device characteristics via machine learning (ML) using a simulation program with integrated circuit emphasis (SPICE) and technology computer-aided design (TCAD) data was considered in cutting-edge technology because of its short computational time and high accuracy [1,2,3,4,5,6,7,8,9,10,11]. The ML-assisted SPICE model is a significantly efficient tool because it can be applied directly for circuit simulation [1,2,3,4]. In terms of design technology co-optimization (DTCO), it can be a powerful tool for improving model hardware correlation and model accuracy [1]. However, with the continuous scaling down in metal–oxide–semiconductor field-effect transistors (MOSFETs), the number of model parameters increased from a few tens to a few hundred [1,2]. The increase in the number of model parameters reduces not only the predictive accuracy but also the physical meaning. Hence, it becomes more difficult and takes longer to extract the parameters with high accuracies using a SPICE model [1]. Although prediction models using SPICE data are advantageous for the fast generation of a large amount of training data, a SPICE compact model is limited by the fact that it cannot include the physics information of numerous devices in the model parameters [1,2]. In this context, TCAD-augmented machine learning (TCAD-ML) is suitable for predicting device characteristics. TCAD data contain a large amount of device information with various physics-based models. Moreover, TCAD-ML can reduce TCAD simulation cases for device design from thousands to tens, which provides faster and lower-cost development cycles without domain expertise [12]. Recent studies using TCAD-ML mostly reported on MOSFETs, such as gate-all-around MOSFETs, FinFETs, and power devices [5,6,7,8,9,10,11]. The prediction of MOSFET characteristics, such as current–voltage (*I-V*) and capacitance–voltage relations, demonstrates that TCAD-ML can provide faster and more accurate outputs to assist with device development than using only TCAD simulations.

Meanwhile, feedback field-effect transistors (FBFETs) attracted great interest regarding their use in next-generation one-transistor memory devices owing to their excellent memory characteristics [13,14]. Nevertheless, given that FBFETs have hysteresis characteristics caused by latch-up phenomena, the prediction of their device characteristics is relatively difficult because of their complex physics, mathematics, and abruptness [13]. In particular, their abrupt switching characteristics are hard to predict because of a complicated relationship between the current gain and various design parameters. Therefore, we proposed a prediction methodology based on TCAD-ML for the device characteristics of silicon nanowire FBFETs using random forest regression. 

In this study, we predicted the full *I-V* characteristics of FBFETs using TCAD-ML with a 400-curve training dataset. Random forest regression, that is, supervised learning, was utilized to achieve high prediction accuracy through its fast and parallel operation and robust performance on finite training data [15]. As a result, we demonstrated that the full *I-V* curves of FBFETs could be deduced from non-simulated device structures and parameters, including device characteristics such as the latch-up voltage (*V*_latch-up_), latch-down voltage (*V*_latch-down_), saturation drain current (*I*_d,sat_), and memory window.

## 2. Materials and Methods

**Figure 1:**
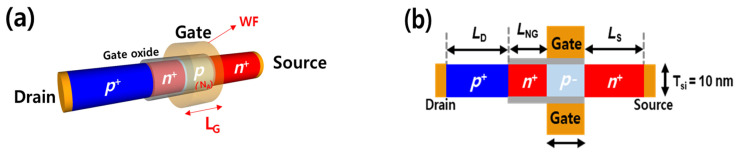
Silicon nanowire FBFET structure for the TCAD simulation: (**a**) three-dimensional structure and randomly varied parameters; (**b**) cross-sectional view of a silicon nanowire FBFET.

Figure 1 shows the device structure of the silicon nanowire FBFET used for the TCAD simulations. A TCAD simulator (Synopsys Sentaurus, Version T-2022.03-SP1) was used to generate the electrical characteristics of the devices. Fundamental device models are included for capturing device characteristics in the TCAD simulations, including the Fermi statistics model, bandgap narrowing model, carrier–carrier scattering, Shockey–Read–Hall recombination, and Auger recombination [16]. To produce an accurate simulation, we applied the mobility model, which included doping dependence mobility, mobility degradation at interfaces, thin layer mobility, and high-field saturation [17]. Furthermore, we considered the scattering of charge carriers by charged impurity ions, which leads to a degradation of carrier mobilities [18]. These physical models are proper to the thin layer device, including nanowire and nanosheet structures [19]. A quasi-stationary numerical TCAD simulation was used to obtain the DC transfer curves of the devices [20]. The TCAD simulations were performed for the forward and reverse voltage sweeps in the *I*_DS_-*V*_GS_ (drain current–gate voltage) transfer curves separately, owing to the hysteresis characteristics. To generate the *I*_DS_-*V*_GS_ transfer curve dataset of the device structure, the gated-channel length (L_G_), doping concentration of the gated-channel region (N_A_), and metal gate work function (WF) were chosen randomly and independently in the ranges 50–250 nm, 10^16^–10^19^ cm^−3^, and 4.7–5.0 eV, respectively, as listed in Table 1. *V*_GS_ was swept from −3.0 to 3.0 V for the forward and reverse voltage sweeps, with approximately 100 intervals. The dimensional parameters included the non-gated channel length (L_NG_), drain length (L_D_), source length (L_S_), thickness of Si (T_Si_), and gate oxide thickness (T_OX_). In this study, L_NG_ = 50 nm, L_D_ = L_S_ = 100 nm, T_Si_ = 10 nm, and T_OX_ = 2 nm, as shown in Figure 1b. A total of 425 *I-V* curves were generated for the training and validation of the TCAD-ML model in the ranges listed in Table 1. A total of 400 *I-V* curves were used for the training dataset, whereas 25 *I-V* curves were reserved for validation. To extract one *I-V* curve from one FBFET device, it took about 600 s on average. This time depended on the mesh size of the device, physical models, and computer specifications. In addition, the total dataset size for the training and validation of the TCAD-ML model was 107,679 × 5, and the training time was less than 500 s.

## 3. Results and Discussion

**Figure 2:**
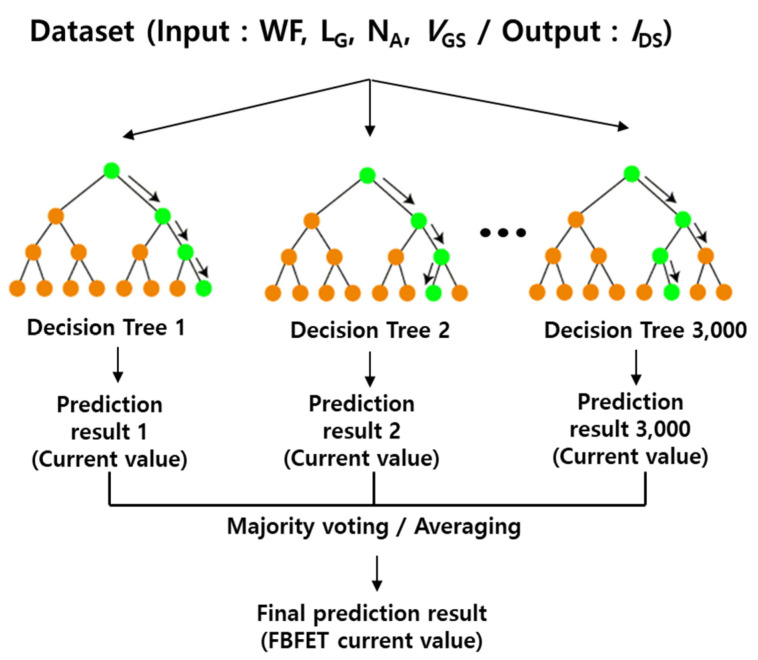
ML framework with a random forest regression algorithm used to predict I_DS_-V_GS_ characteristics. A total of 3000 decision trees were used in the experiments.

The ML framework with random forest regression is shown in Figure 2. Random forest regression models are faster and more accurate than boosted regressions [21]; therefore, we selected this model for the prediction of device characteristics. In the TCAD-ML model, device characteristics were predicted using linear regression, neural network, and random forest regression algorithms [22,23,24,25]. However, linear regression has limitations for the prediction of nonlinear dependency despite its simple design [22]. Moreover, a neural network introduces more design complexity and longer computational time for non-linear regression. However, random forest regression provides less complexity and higher computation speed for non-linear problems due to its simple tree-structured black-box design [22]. In particular, a random forest regression model captures the relationship between the device characteristics and design parameters well [22,23]. In addition, to predict the device characteristics, random forest regression provided higher accuracy and a low error rate than linear regression, multiple layer perceptron, and multi-take learning in our previous research [26,27]. Therefore, random forest regression is appropriate to use to predict device characteristics due to its fast, robust, and parallel process [28]. To train the machine for the particular device characteristics of FBFETs, the random forest regression model was implemented for the majority voting to be executed by the prediction of all the trees by means of the output. In our ML framework, the four device parameters (L_G_, N_A_, WF, and *V*_GS_) were input features to the decision trees, and the drain current *I*_DS_ constituted the output, as shown in Figure 2. The device design parameters, such as WF, L_G_, and N_A_, are the most important parameters for designing FBFETs [29,30,31]. Hence, we selected these input parameters. For training, a total of 3000 decision trees were used. The TCAD-ML model was learned from 400 *I-V* curves obtained from 400 FBFETs, and then we predicted the drain current values corresponding to gate voltages in 25 *I-V* curves obtained from 25 FBFETs, which composed the validation dataset. 

**Figure 3:**
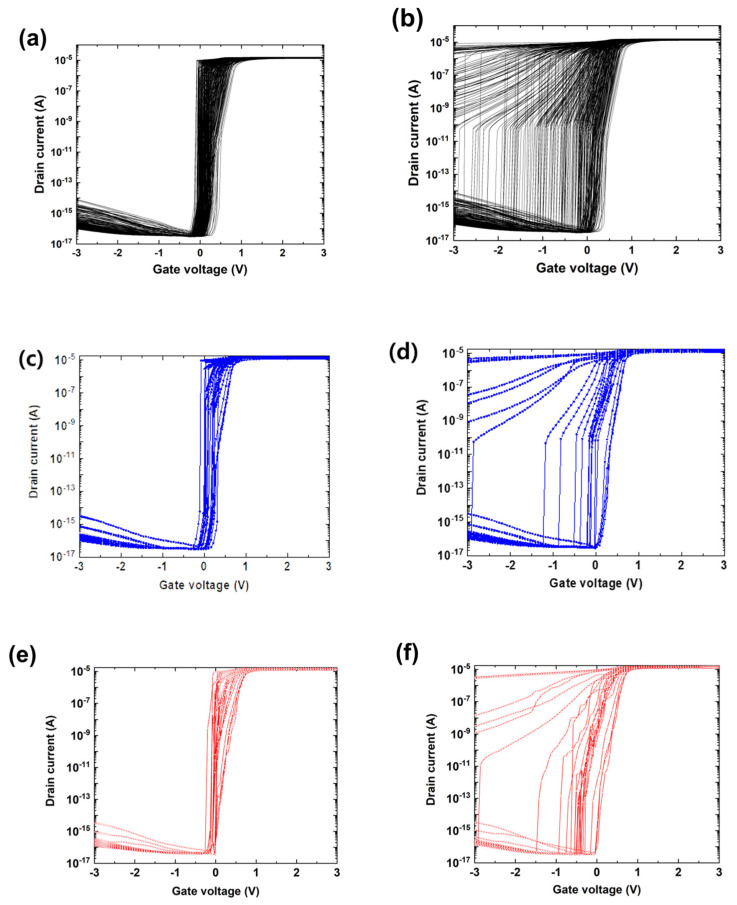
Training dataset with 400 *I_DS_-V_GS_* transfer curves (**a**,**b**) and the validation dataset with 25 *I_DS_-V_GS_* transfer curves (**c**–**f**). (**a**) *I_DS_-V_GS_* transfer curves for a forward gate voltage sweep from −3.0 to 3.0 V. (**b**) *I_DS_-V_GS_* transfer curve in a reverse gate voltage sweep from 3.0 to −3.0 V. (**c**) Forward-sweep *I_DS_-V_GS_* transfer curves simulated using the TCAD simulator. (**d**) Reverse-sweep *I_DS_-V_GS_* transfer curves predicted using our TCAD-ML algorithm. (**e**) Forward-sweep *I_DS_-V_GS_* transfer curves simulated using the TCAD simulator. (**f**) Reverse-sweep *I_DS_-V_GS_* transfer curves predicted using our TCAD-ML algorithm.

Figure 3 shows the entire training dataset in terms of the *I_DS_-V_GS_* transfer curves for the forward (a) and reverse (b) voltage sweeps. Once the machine was trained, we fed the TCAD-ML model with three of the device parameters (L_G_, N_A_, and WF) and gate voltages, and predicted the drain current. Figure 3c,d show the validation dataset. Figure 3e,f show the predicted results for the forward and reverse voltage sweeps. We achieved high prediction accuracy with large R-squared (R^2^) values, i.e., 0.9938 and 0.9953, for the forward and reverse voltage sweeps, respectively. Furthermore, the low prediction error rates with root-mean-square errors (RMSEs) equal to 0.0471 and 0.0273 were attained for the forward and reverse voltage sweeps, respectively. The TCAD-ML model could reconstruct the shape of the *I-V* transfer curves while capturing a diversity of the device features, such as the latch-up and latch-down voltages. Particularly, it could capture the latch-up (latch-down) voltages at which positive feedback loop phenomena were generated (diminished), as shown in Figure 3e (Figure 3f).

**Figure 4:**
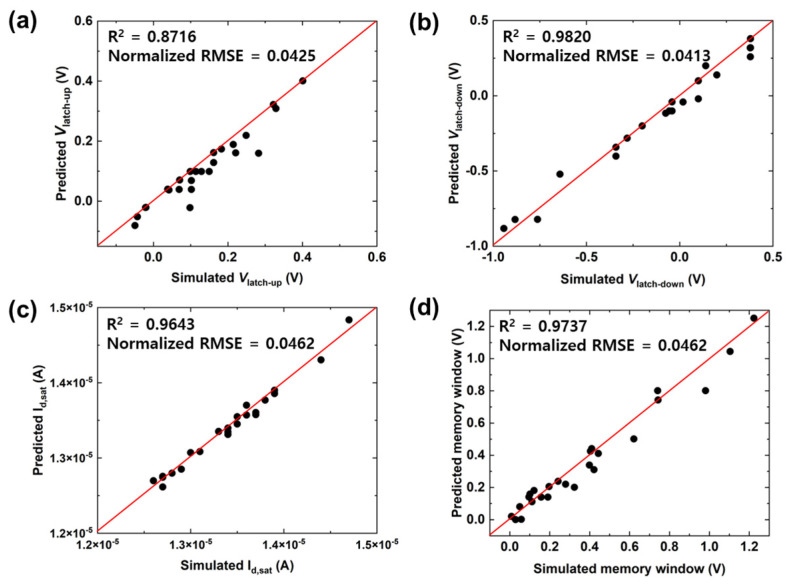
Scatter plots showing the prediction of (**a**) *V*_latch-up_, (**b**) *V*_latch-down_, (**c**) *I*_d,sat_, and (**d**) memory window, which were the major extracted parameters from the predicted *I*_DS_-*V*_GS_ transfer curves. *V*_latch-up_ and *V*_latch-down_ were defined as the gate voltages at *I*_DS_ = 10^−9^ A. *I*_d,sat_ was defined as the drain current at *V*_GS_ = 3.0 V. The memory window was defined as the voltage difference between *V*_latch-down_ and *V*_latch-up_.

It is particularly important to predict not only the full *I-V* curves but also the device characteristics that determine the device operation. To operate FBFETs as memory devices, the important features in their *I-V* transfer curves are *V*_latch-up_, *V*_latch-down_, *I*_d,sat_, and the memory window. Figure 4 shows the scatter plots of *V*_latch-up_, *V*_latch-down_, *I*_d,sat_, and the memory window extracted from the predicted full *I-V* transfer curves. We could predict the aforementioned features with R^2^ values equal to 0.8716, 0.9820, 0.9643, and 0.9737, respectively. The prediction of *V*_latch-up_ was relatively more difficult than the other predictions because of the abrupt increase in the drain current. Despite the low R^2^ value of 0.8716 for *V*_latch-up_, we could predict the trend of *V*_latch-up_, as shown in Figure 4a. Furthermore, given that the TCAD-ML model properly captured the relationship between the variable parameters and the output, *V*_latch-down_ and *I*_d,sat_ were predicted with high accuracy. The prediction accuracy of the memory window depended on the prediction accuracy of *V*_latch-down_, given that the *V*_latch-down_ variability was greater than that of *V*_latch-up_, as shown in Figure 4d; note that the memory window was defined as the voltage difference between *V*_latch-up_ and *V*_latch-down_. Therefore, we demonstrated that 400 full *I-V* curves were sufficient to predict the device characteristics (*V*_latch-up_, *V*_latch-down_, *I*_d,sat_, and the memory window) using the ML model.

The prediction accuracies and error rates of the device characteristics are listed in Table 2 and Table 3, respectively, for training datasets ranging from 25 to 400 curves. *V*_latch-up_ and *I*_d,sat_ were extracted using the *I-V* transfer curves in the forward voltage sweep, whereas *V*_latch-down_ was extracted using the *I-V* transfer curves in the reverse voltage sweep. For the 25-curve dataset, it was difficult to learn the relationship between the variable parameters and the device characteristics for *V*_latch-up_ and *I*_d,sat_. In particular, for the 25-curve and 50-curve datasets, *I*_d,sat_ had a negative R^2^ value, implying that the model did not follow the trend of the dataset. A recent study concluded that training is possible with only 25-curve datasets for MOSFET full *I-V* curves and device characteristics, such as *I*_d,sat_, the off current, drain-induced barrier-lowering, and transconductance [6]. Given that MOSFETs have more continuous and smooth characteristic functions for their *I-V* transfer curves over all their operating regions than those of FBFETs, their *I-V* transfer curves can be predicted using datasets composed of 25 curves or fewer. However, for FBFETs, predicting the *I-V* transfer curves is relatively difficult owing to the abrupt current increase and complex physics. Hence, for the *I-V* transfer curves in the forward voltage sweep, at least a 400-curve training dataset was required for the prediction of the device characteristics. In our previous research on the FBFET design, *V*_latch-up_ had a complex relationship between L_G_, L_NG_, L_D_, L_S,_ N_A_, and T_OX_ [13]. In particular, L_G_ and N_A_ directly affected the current gain of FBFETs, which determined *V*_latch-up_. Therefore, as the number of the training dataset increased from 25 to 400, the prediction accuracy gradually increased while the TCAD-ML model captured the relationship between *V*_latch-up_ and input design parameters from 0.0110 to 0.8716. This prediction showed that WF, L_G_, and N_A_ could represent *V*_latch-up_, *V*_latch-down_, *I*_d,sat_, and the memory window, which are the representative device characteristics of FBFETs. In addition, to improve the prediction accuracy and error rate with the same number of training sets, an increase in the number of *V*_GS_ values should be required. In contrast, for the *I-V* transfer curves in the reverse voltage sweep, learning is possible with a training dataset containing at least 100 curves. Therefore, thousands of TCAD simulation cases are generally required for device development. Nevertheless, the TCAD simulation cases can be reduced to a set of 100–400 cases. In particular, to predict total forward- and reverse-sweep *I_DS_-V_GS_* transfer curves, including major parameters, the TCAD-ML model reduced the device development time by a factor of 8. For the estimation of the computational time for the device development, a computational time of 600 s was assumed for extracting one curve from one FBFET device. Then, based on the training time of the TCAD-ML model, the total training time using the random forest regression was assumed to be 500 s. In general, as over a thousand TCAD simulations are required for device development, we can expect to reduce the computational time for device development by approximately 80% for the forward voltage sweep, and about 95% for the reverse voltage sweep compared with the conventional computational time. Our study demonstrated that the prediction of the FBFET characteristics by training machines is possible with limited training data and reduced computational time. 

In this study, we demonstrated that the TCAD-ML model could predict the full *I-V* curve of FBFETs with abrupt switching characteristics. We focused on the feasibility of the prediction of the major device characteristics, including *V*_latch-up_, *V*_latch-down_, *I*_d,sat_, and the memory window, as well as the relationship between the number of training sets and prediction accuracy. Therefore, this research acts as a basis for the development of FBFET TCAD-ML integrated models.

## 4. Conclusions

In this study, we demonstrated that the ML prediction of the full *I-V* curves of FBFETs with limited TCAD simulation datasets was possible. The proposed TCAD-ML model enabled the fast and accurate prediction of the device characteristics (including *V*_latch-up_, *V*_latch-down_, *I*_d,sat_, and the memory window) of FBFETs. The computational time of the proposed TCAD-ML model was much less than that of a conventional TCAD simulation. Therefore, the TCAD-ML model is expected to accelerate the development of novel device technologies through its notably higher simulation efficiency.

## Figures and Tables

**Table 1:** TCAD simulation parameters.

Name	Unit
Gate work function (WF)	4.7–5.0 eV
Gated-channel length (L_G_)	25–250 nm
Gated-channel region doping concentration (N_A_)	10^15^–10^19^ cm^−3^
Gate voltage (*V*_GS_) sweep range	−3.0–3.0 V
Non-gated channel length (L_NG_)	50 nm
Drain length (L_D_)	100 nm
Source length (L_S_)	100 nm
Gate oxide thickness (T_OX_)	2 nm
Nanowire thickness (T_Si_)	10 nm
Drain region doping concentration	1 × 10^19^ cm^−3^
Source region doping concentration	1 × 10^19^ cm^−3^
Non-gated channel region doping concentration (N_D_)	1 × 10^19^ cm^−3^

**Table 2:** Prediction accuracy as a function of the number of *I-V* curves in the dataset for the prediction of device characteristics.

	Prediction Accuracy (R^2^)			
Number of Training Curves	*V*_latch-up_	*V*_latch-down_	*I*_d,sat_	Memory Window
25	0.0110	0.4480	−0.7819	0.5983
50	0.4427	0.7158	−0.3019	0.7284
100	0.4464	0.9265	0.3801	0.728
150	0.4832	0.9382	0.3437	0.7398
200	0.5709	0.9539	0.9187	0.8685
300	0.7301	0.9676	0.9466	0.9101
400	0.8716	0.9820	0.9643	0.9737

**Table 3:** Error rate as a function of the number of *I-V* curves in the dataset for the prediction of device characteristics.

	Normalized RMSE			
Number of Training Curves	*V*_latch-up_	*V*_latch-down_	*I*_d,sat_	Memory Window
25	0.2695	0.1966	0.3289	0.1627
50	0.2023	0.1410	0.2811	0.1338
100	0.2016	0.0717	0.1940	0.1616
150	0.1948	0.0676	0.2000	0.1309
200	0.1763	0.0567	0.0717	0.0972
300	0.1027	0.0501	0.0583	0.0570
400	0.0425	0.0413	0.0462	0.0462

## Data Availability

The data presented in this study are available on request from the corresponding author.
